# Extending the ‘host response’ paradigm from sepsis to cardiogenic shock: evidence, limitations and opportunities

**DOI:** 10.1186/s13054-023-04752-8

**Published:** 2023-11-27

**Authors:** Marie Buckel, Patrick Maclean, Julian C. Knight, Patrick R. Lawler, Alastair G. Proudfoot

**Affiliations:** 1 Department of Perioperative Medicine, Bart’s Heart Centre, St. Bartholomew’s Hospital, London, UK; 2 Wellcome Centre for Human Genetics, University of Oxford, Oxford, UK; 3 Chinese Academy of Medical Sciences Oxford Institute, University of Oxford, Oxford, UK; 4 Peter Munk Cardiac Centre, University Health Network, University of Toronto, Toronto, ON Canada; 5 McGill University Health Centre, McGill University, Montreal, QC Canada; 6 Queen Mary University of London, London, UK

**Keywords:** Cardiogenic shock, Sepsis, Critical illness, Inflammation, Host response, Immune dysregulation

## Abstract

Recent clinical and research efforts in cardiogenic shock (CS) have largely focussed on the restoration of the low cardiac output state that is the *conditio sine qua non* of the clinical syndrome. This approach has failed to translate into improved outcomes, and mortality has remained static at 30–50%. There is an unmet need to better delineate the pathobiology of CS to understand the observed heterogeneity of presentation and treatment effect and to identify novel therapeutic targets. Despite data in other critical illness syndromes, specifically sepsis, the role of dysregulated inflammation and immunity is hitherto poorly described in CS. High-dimensional molecular profiling, particularly through leukocyte transcriptomics, may afford opportunity to better characterise subgroups of patients with shared mechanisms of immune dysregulation. In this state-of-the-art review, we outline the rationale for considering molecular subtypes of CS. We describe how high-dimensional molecular technologies can be used to identify these subtypes, and whether they share biological features with sepsis and other critical illness states. Finally, we propose how the identification of molecular subtypes of patients may enrich future clinical trial design and identification of novel therapies for CS.

**Graphical Abstract:**

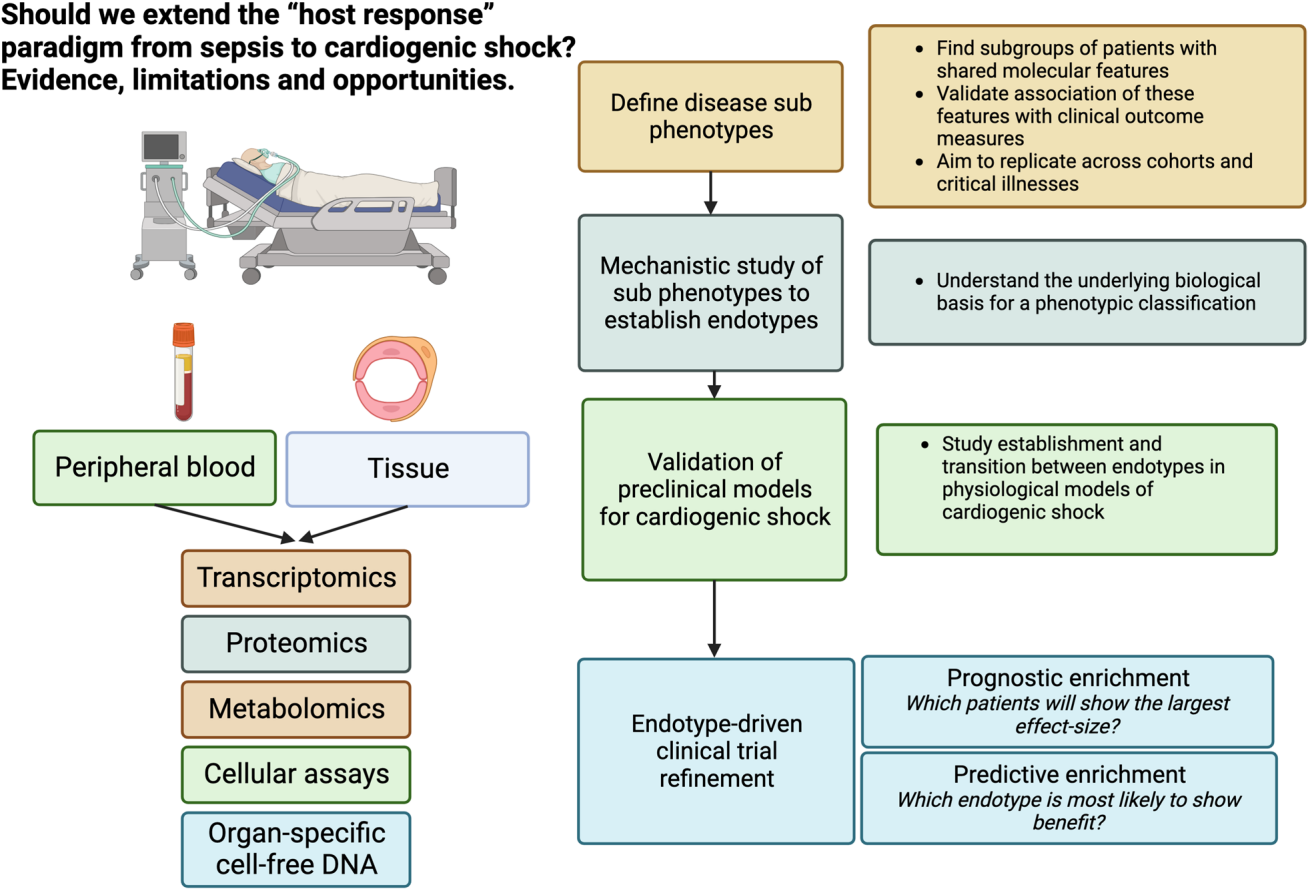

## Introduction

Cardiogenic shock (CS) is a complex syndrome of hypoperfusion resulting from cardiac dysfunction. Historically, a simple model of the pathophysiology of CS has focused on a reduction in cardiac output leading to reduced end-organ perfusion with systemic tissue oxygen starvation which culminates in death in 30–50% of patients [1]. As such, beyond specific interventions such as culprit-vessel percutaneous coronary intervention (PCI) in acute myocardial infarction-CS (AMI-CS), current best care is supportive, targeting normalisation of hemodynamic (cardiac output and blood pressure) and biochemical perturbations through the use of inopressors and/or mechanical circulatory support (MCS) [2, 3]. However, augmentation of cardiac output has yet to demonstrate a survival benefit in clinical trials [4–7]. Recently published randomised control trial (RCT) [7] and meta-analysis [8] data illustrate no mortality benefit with the use of MCS in CS. This highlights the importance of finding alternative or complementary therapeutic strategies which have the potential to modify the syndrome itself once established.

One of the many challenges that CS presents, and in parallel with other critical illness syndromes such as sepsis [9, 10], is significant heterogeneity. The magnitude and haemodynamic presentation of shock differs considerably between patients, contributing to a range of clinical phenotypes, often despite similar aetiological insults [11]. Further heterogeneity is observed in the individual response to supportive measures which likely impacts disease severity, prognosis and treatment response. Whilst this variation has been described clinically [11–13], its pathobiological basis has hitherto been poorly delineated. In parallel with the response to infection in sepsis, cardiomyocyte necrosis and tissue hypoxia in CS may activate an inflammatory response. Although intended to be reparative and restorative, as in sepsis and ARDS, it is plausible that the ‘host response’ to myocardial injury and end organ failure may be misaligned, maladaptive or even injurious. Potential adverse sequelae of maladaptive inflammatory and immune activation include impaired microcirculation, inappropriate vasodilatation further compromising tissue perfusion, impaired cardiomyocyte recovery and compounded organ hypoperfusion through inappropriate vasodilatation and progression of the shock state. Importantly, the extent of inflammatory activation in response to AMI varies from patient-to-patient [14] with greater inflammation linked to short- and longer-term adverse outcomes [15]. This emerging ‘host-response’-oriented model is now established in inflammatory critical illness but has yet to be fully appreciated in CS. Accordingly, in this review we consider the relevant basis for such a model in CS, focusing primarily on AMI-CS where the pathophysiology and outcomes have been better studied to date. We theorise how this may enhance current research endeavours to improve patient outcomes.

## Cardiogenic shock subphenotypes

In recognition of a need to relate clinical care and triage with CS severity, recent societal and registry efforts have attempted to stratify patients into groups defined by clinical, haemodynamic, metabolic and biochemical parameters [11–13]. The Society for Cardiovascular Angiography and Interventions (SCAI) Shock Classification, proposed by an expert panel, groups patients into stages A (“At risk”) to E (“Extremis”) [12]. The SCAI classification is currently the most well-validated in terms of mortality prediction [16–19] and has been adopted into clinical guidelines and clinical practice [20, 21]. Extending this, an unsupervised machine learning analysis of registry data identified three distinct clinical CS phenotypes, termed ‘non-congested’, ‘cardiorenal’ and ‘cardio-metabolic’ [11]. These phenotypes had distinct haemodynamic and biochemical profiles with reproducible associations with shock severity and mortality [22].

Whilst such groupings may inform CS severity, there is evidence that the patients defined within these cohorts, their associated mortality risk and treatment responses remain highly heterogeneous [23–25]. These classifications also provide no additional information on pathobiology to guide treatments. Delineating patient groups based on combinations of clinical manifestations and biomarker patterns that link with disease pathobiology, so-called sub-phenotyping, has been proposed to bridge underlying disease mechanisms with clinical phenotype [26]. Notably, in the recent ‘Extra-Corporeal Life Support in Infarct Related Shock’ (ECLS-SHOCK) study of 420 patients with AMI-CS, mortality was approximately 50% and of those that died, more than half died of refractory cardiogenic shock [7]. This finding suggests that there are drivers of persistent shock and organ failure that extend beyond cardiac output. Vasodilatory CS is defined by a haemodynamic profile characterised by low systemic vascular resistance and reduced cardiac output (CO) with or without infection. This subphenotype appears to have greater shock severity, more organ failures and a worse prognosis [27, 28]. Similarly, patients with microcirculatory dysfunction, or the uncoupling of macro- and peripheral microcirculatory blood flow in CS, may represent an additional sub-phenotype reflecting immune-mediated endothelial dysfunction and microvascular thrombosis identifiable by impairments in capillary refill time [29, 30].

## The ‘host response’ to cardiogenic shock

The role of inflammation in cardiovascular disease [31] and the immune response to myocardial infarction is well established [32, 33]. Data in CS are largely derived from small-scale observational studies or sub-studies of randomised controlled trials examining circulating cytokines and proteins, with limited mechanistic data. Higher levels of biomarkers of systemic inflammation, such as C-reactive protein (CRP), procalcitonin (PCT) and IL-6, are associated with more severe hypoperfusion in CS, whilst levels of PCT and IL-6 correlate with multi-organ failure (MOF) [34] and mortality [35]. In a small single-centre study of AMI-CS, admission IL-6 was more strongly associated with 30-day mortality than more traditionally cardiac-specific markers, such as N-terminal pro-brain natriuretic peptide (NT-pro-BNP) [36]. Table 1 presents a comprehensive, though not exhaustive, list of inflammatory biomarkers implicated in the pathophysiology of CS.

**Table 1:** Studies investigating the role of inflammatory mediators in CS

Author	Cohort	Inflammatory biomarkers	n	Main findings
Geppert [34]	Mixed CS vs SS vs non-critically ill patients Single centre	IL-6	88	Higher IL-6 in CS patients with MOF vs CS without IL-6 > 200 pg/ml 93% specificity & 100% sensitivity for prediction of MOF
Geppert [37]	Mixed CS vs SS vs non-critically ill patients Single centre	CRP and PCT	66	Median PCT elevated in CS patients vs non-critically ill controls, but not as high as those with SS PCT > 2 g/ml in CS correlated with organ failures
Debrunner [38]	AMI vs AMI-CS Single entre	TNF-alpha; IL-6; IL1Ra	41	AMI-CS had significantly higher cytokine levels. IL-1Ra elevated early in course of AMI-CS
Picariello [39]	CS following STEMI vs STEMI vs NSTEMI Single centre	PCT, CRP	52	CRP reflects degree of myocardial ischaemia. PCT reflective of higher degree of inflammatory activation, being positive in all CS patients
Prondzinsky [40]	AMI-CS Single centre	IL-6, -7,-8, -10; IL-1β	40	IL-6, -7, -8 and -10 predicted mortality
Prondzinsky [41]	AMI-CS Single centre	INF-gamma, TNF-alpha, MIP-1beta, G-CSF, MCP-1beta	40	Patients with elevated pro and anti-inflammatory cytokine levels had a higher risk of dying. Maximal levels are also suited to identifying survivors
Andrié [36]	AMI-CS Single centre	IL-6, PCT, NT-pro-BNP	87	Multivariate analysis demonstrated that admission IL-6 had the highest level of significance in predicting 30-day mortality
Fuernau [42]	AMI-CS Multi centre	GDF-16; Osteoprotegerin	190	Levels of GDF-15 on admission is an independent predictor of 30-day mortality
Lipkova [43]	AMI-CS vs AMI Single centre	RANTES	173	Lower serum RANTES in AMI-CS vs AMI
Liu [44]	MIxed-CS requiring ECMO Single centre	IL-6, -8, -10, MCP-1, TNF alpha, IL-1beta, Prdx1	46	Prdx1 peaked earlier than other cytokines and a higher initial plasma level predicted a worse outcome
Parenica [45]	AMI-CS Single centre	CRP, PCT, Presepsin, PTX3	80	CRP and PCT both elevated but did not discriminate the presence of concomitant infection 12-h PTX3 highest AUC for 3-month mortality prediction
Takagi [46]	Mixed CS Multi centre	cDPP3	57	Association between levels of cDPP3 > 51.9 ng/ml & 90-day mortality. Delta cDPP3 within first 24 h correlated with mortality
Cuinet [47]	Mixed CS Single centre	Differential WBC counts; IL-1β, IL-5, IL-6, IL-10, TNF-α, IFN-γ, MCP-1 and eotaxin (CCL11)	24	Elevated levels of IL-6, IL-10 and MCP-1 correlate with shock severity Patients with most severe shock have reduced lymphocyte and monocyte counts at 48 h and 6–8 days post-admission
Kataja [35]	Mixed CS Multi centre	IL-6; PCT; CRP; GDF-15	183	Elevated serum PCT & IL-6 associated with clinical & biochemical signs of hypoperfusion, organ dysfunction & mortality
Büttner [48]	AMI-CS Multi centre	SelP	147	Levels > 75th percentile at day 3 significantly correlated with 30-day mortality
Jentzer [49]	CICU population Multi-centre registry	NLR	8280	Elevated NLR associated with worse outcomes across shock severities
Roth [50]	Mixed CS supported with VA-ECMO Single centre	NLR; PLR and PCT	92	NLR independently associated with mortality
Dudda [51]	Mixed CS Single centre	CRP, WBCs	240	WBC of > 10 × 106/ml on admission and a > 200% increase in CRP between days 1 and 3 associated with increased 30-day mortality
Wenzl [52]	AMI + AMI CS Multi-centre	cDPP3	4787 213 (AMI-CS)	High levels of cDPP3 independently associated with increased risk of development of in-hospital CS, with a dose–response relationship

AMI = acute myocardial infarction; CICU = cardiac intensive care unit; CS = cardiogenic shock; cDPP3 = circulating dipeptidyl peptidase 3; CRP = C-reactive protein; G-CSF = granulocyte-colony-stimulating factor; GDF-15 = growth-differentiation factor-15; IFN-gamma = interferon gamma; IL-1β = interleukin- 1; IL-1Ra = interleukin-1 receptor antagonist; IL-5: interleukin-5; IL-6 = interleukin-6; IL-7 = interleukin-7; IL-8 = interleukin-8; IL-10; interleukin-10; MIP-1beta = macrophage inflammatory protein-1beta; MCP-1beta = monocyte chemoattractant protein-1beta; MOF = multi-organ failure; NLR = neutrophil to lymphocyte ratio; NSTEMI = non-STelevation myocardial infarction; NT-pro-BNP = N-terminal pro-brain natriuretic peptide; PTX 3 = pentraxin 3; Prdx-1 = peroxiredoxin-1; PLR = platelet to lymphocyte ratio; PCT = procalcitonin; RANTES = regulated on activation, normal T cell expressed and secreted; SelP = selenoprotein P; SS = septic shock; STEMI = ST-elevation myocardial infarction; TNF-alpha = tumour necrosis factor-alpha; VA-ECMO = venoarterial extracorporeal membrane oxygenation; WBC = white blood cells

The occurrence of sepsis super-imposed on CS, and CS secondary to sepsis (septic cardiomyopathy), further drives the hypothesis that dysregulated immunity may contribute to CS pathobiology [53–55]. Patients with CS have multiple risk factors for increased risk of infection including preceding cardiac arrest, gut hypo-perfusion and risk of bacterial translocation, use of multiple invasive central venous catheters and prolonged mechanical ventilation and intensive care unit (ICU) stay. Estimates of the incidence of concomitant or secondary sepsis range widely, from 6% of patients with AMI-CS in a large US payer database to around 50% in single-centre observational cohort studies [45, 55]. Reasons for this wide range likely relate to overlap between the traditional clinical and biochemical markers used to diagnose CS and sepsis [45, 53, 54] as well as variability in the requirement for detection of a causative microorganism. The presence of concomitant sepsis is associated with increased shock severity [28], an increased risk of organ failures [55, 56] and higher mortality [45, 55].

Whether infection is a driver of dysregulated immunity or secondary to it, however, remains unclear. One potential source of sterile inflammation is endotoxin translocation from either digestive tract hypoperfusion or ischemia–reperfusion in CS. Endotoxemia reduces cardiac performance [57] and can propagate a low cardiac output state [58]. Despite sound rationale, three small studies [59–61] have failed to identify direct proof of endotoxemia in patients with CS with the caveat that no study has hitherto reported quantitative measurement (i.e. mass) of circulating endotoxins and many endotoxin assays have inherent detection limitations. An alternative explanation for the occurrence of secondary sepsis in AMI-CS is the development of maladaptive immune response, or even an acquired immunodeficiency. In a large cohort of critically ill patients with diffuse insults, after an initial phase of adaptive injury-induced immune response, a persistence of altered T-cell and monocyte response at one week was associated with secondary infection [62].

Septic cardiomyopathy typifies the overlap between septic and cardiogenic shock. Similar to CS, the precise mechanisms of cardiac dysfunction remain poorly elucidated. There is overlap in the observed immune response [38, 44, 47, 63, 64] to both AMI-CS and septic cardiomyopathy, despite the absence of a primary cardiac insult in the former. It is postulated that the systolic dysfunction of septic cardiomyopathy is an adaptive response which manifests as more classical cardiogenic shock when there is maladaptation and associated cellular dysfunction [65, 66]; a comprehensive review is beyond the scope of this article but is covered elsewhere [67]. Further research into potentially shared pathobiology between septic cardiomyopathy and cardiogenic shock syndromes through comparisons of existing multi-modal sepsis datasets with emerging CS datasets will hopefully be mechanistically and therapeutically revealing.

An emerging potential modulator of CS pathobiology is the circulating enzyme dipeptidyl peptidase 3 (DPP3). This zinc-dependent metallopeptidase is found intracellularly throughout all the body’s organ systems [68] and is released into the circulation during cell death [69, 70]. High levels of circulating DPP3 (cDPP3) have been found to be associated with increased severity in a range of shocked states [71]. Deletion of DPP3 impacts production of both proinflammatory and anti-inflammatory cytokines [72]. Injection of DPP3 in a murine model produced myocardial depression, whilst administration of a specific antibody targeted against cDPP3 normalised haemodynamics [73]. In vivo cDPP3 levels were measured in patients recruited to the multi-centre Optima CC trial [46] comparing epinephrine versus norepinephrine for haemodynamic support in CS [74]. High levels of cDPP3 (> 51.9 ng/ml) were associated with greater risk of death at 90 days, greater organ dysfunction and lower cardiac index, findings which have been confirmed in subsequent observational studies [75, 76]. A large multi-centre, prospective study investigating the role of cDPP3 in acute coronary syndromes (ACS) found that high levels were independently predictive of the development of in-hospital CS (HR 1.49, 95% CI 1.14–1.96, *P* = 0.004) [52]. Whilst promising, further data to clarify the precise biological role of DPP3 in CS are needed.

The contribution of baseline inflammation in the system-wide pathogenesis of CS has been further highlighted by a potential role for clonal haematopoiesis (CH) [77, 78]. CH is the acquisition of somatic mutations of potentially oncogenic genes in haematopoietic stem cells (HSC) that results in distinct immune cell clones with dysregulated function. The carriage of these mutations has been associated with increased risk of atherosclerotic cardiovascular disease and cardiac failure [79]. Some in vitro studies have suggested that macrophages and monocytes with clonal features have a hyper-inflammatory phenotype [79, 80]. In a biomarker sub-study of the multi-centre *“Culprit Lesion Only PCI Versus Multivessel PCI in Cardiogenic Shock—CULPRIT-SHOCK”* trial [81] a correlation between the burden of CH and risk of death or requirement for renal replacement therapy was observed [77]. CH was also associated with increased levels of the inflammatory cytokines IL-6 and IL1-beta. Similarly, in a single-centre matched retrospective cohort study comparing the presence of CH in patients with CS versus those with stable heart failure (HF), CS patients had a 50% higher prevalence of CH-related mutations (odds ratio 1.5; p = 0.02) [78]. Higher CH was associated with reduced survival and dysregulation of circulating inflammatory cytokines, particularly in those patients with mutations of tet methylcytosine dioxygenase 2 (*TET2*). Collectively these data suggest that CH may augment the acute inflammatory state in CS, contributing to the development of superimposed vasodilatory shock, and in turn to worse outcomes.

**Fig. 1:**
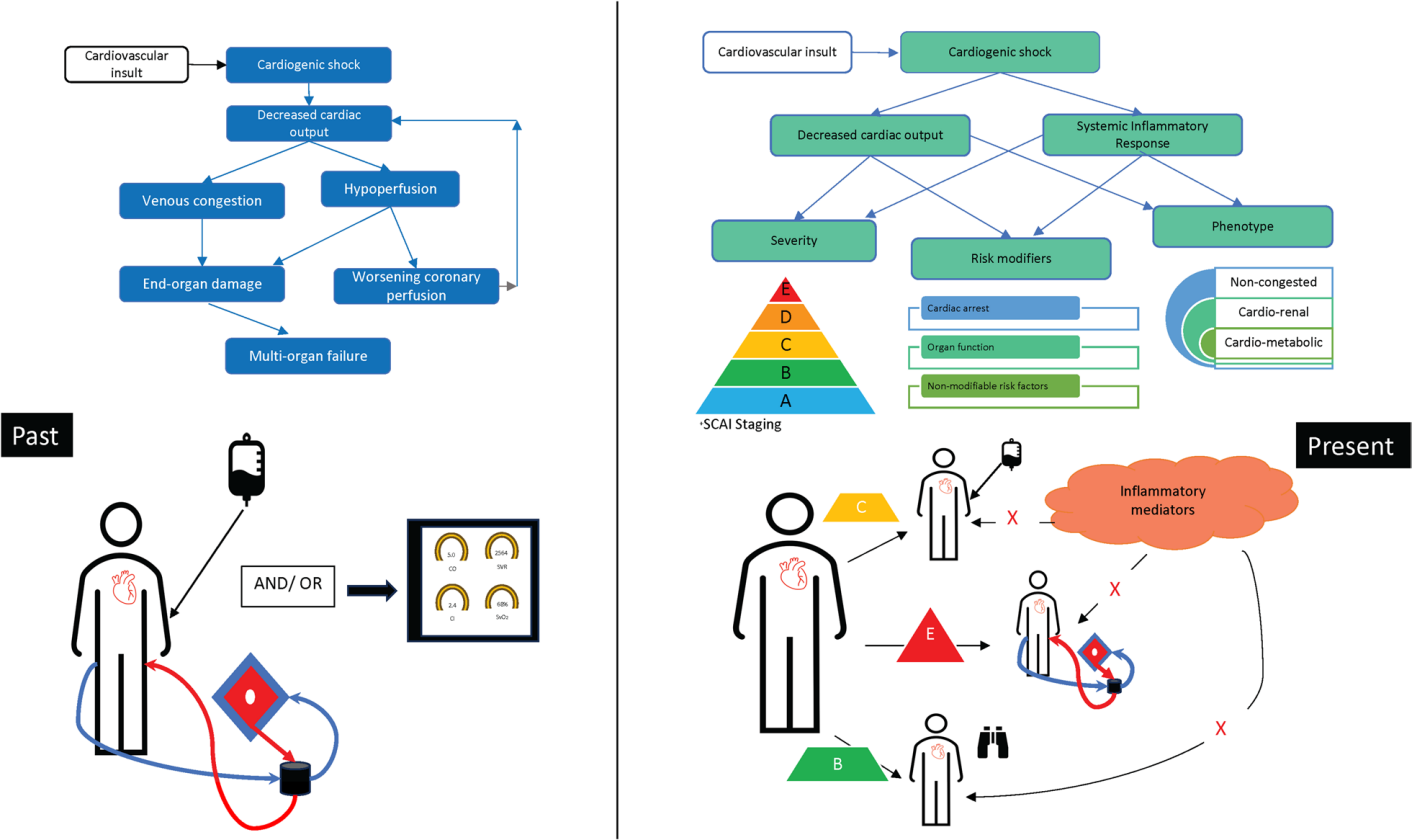
Past and present risk stratification in CS. + Severity staging pyramid adopted from Society of Cardiovascular Angiography & Intervention (SCAI) [12]. Historically the management of cardiogenic shock (CS) has focussed on the normalisation of haemodynamic and biochemical parameters. As such, early research has compared the optimal modality to achieve this i.e. pharmacotherapy versus mechanical circulatory support. More recently, investigators have aimed phenotype patients into risk categories using both clinical parameters and measurements of inflammatory mediators to define severity of shock or risk of death

Given the assertion that immune activation and dysregulated inflammation are implicated in the pathogenesis of CS, it would follow that immunomodulation may improve clinical outcomes. To date, despite trials in patients with heart failure [82–84], there have been no clinical trials specifically testing immunomodulatory therapy in patients with CS. This likely reflects both challenges of studies in CS patients per se as well as the rudimentary state of understanding of the inflammatory response as a therapeutic target. One ongoing study will assess the effects of the IL-6 monoclonal antibody tocilizumab on the development of CS after MI (ClinicalTrials.gov Identifier: NCT05350592), testing the importance of inflammation and the neurohormonal response to the development of CS. Another ongoing study will test the Oxiris membrane™, which removes circulating pro-inflammatory cytokines and lipopolysaccharides (ClinicalTrials.gov Identifiers: NCT05642273 and NCT04886180), in the most severe cohort of CS patients supported with venoarterial extracorporeal membrane oxygenation. Figure 1 illustrates how the past and current understanding of CS pathophysiology has shaped both treatment goals and clinical trial design.

### Future State of the ‘Host Response’ to Cardiogenic Shock: sub-phenotypes, endotyping and treatable traits

The observations describing the inflammatory response to CS above highlight the need for future studies which capture rich data on immune phenotypes. The immune system is highly complex, characterised by multidimensional relationships across multiple scales. Genes, cells and whole organs function together to preserve homeostasis often with multiple layers of redundancy. This complexity has driven the use of high-dimensional readouts, based on genetics, transcriptomics (RNA-sequencing), proteomics and metabolomics, to collect measurements informative for different features of the host immune state. In CS, there is the opportunity to discover molecular features or mechanisms associated with observed clinical traits, either individually in the form of single associated genetic loci, genes or proteins, or in ensemble gene sets or co-expression modules. Such molecular features, as well as clinical patient characteristics, can enable the discovery and definition of sub-phenotypes (subgroups). A disease endotype refers to instances where sub-phenotype (subgroup) characteristics/biomarkers define or associate with a specific pathophysiological mechanism. In terms of clinical utility, there is interest in establishing treatable traits, whereby sub-phenotype characteristics/biomarkers identify a group of patients with a specific pathophysiological derangement which manifests a predictable response to a specific therapy.

The most common approach to sub-phenotype and endotype discovery is peripheral blood leukocyte transcriptomics using RNA-sequencing. Whole blood provides a snapshot of the gene expression and abundance of cell types at different stages of haematopoiesis. Whilst peripheral blood sampling is logistically simple, the cellular composition of peripheral blood may change significantly over the natural history of critical illness and in response to critical care interventions and therapies [85]. Whole blood isolation of peripheral blood mononuclear cells (PBMCs) is informative but limits analysis to lymphocyte and monocyte populations [14]. This approach has the advantage of isolating cell types with greater relevance to the adaptive immune system but is relatively laborious and omits the key granulocyte populations which are the major effectors of the acute host response. The granularity of RNA-sequencing data acquisition and cellular heterogeneity can be further enhanced by quantification of the transcriptome of individual cells (single-cell RNA-sequencing) as opposed to measurement of average gene expression measured across a large population of cells (bulk RNA-sequencing).

The largest whole-blood transcriptomic studies, predominantly performed in sepsis populations [86–89], have used unsupervised clustering approaches to partition patients into discrete categories. The hypothesis is that these gene-expression groupings reflect fundamental differences in the host response, which are not better explained by known clinical covariates like age, sex, blood cell proportions, causative organism or immunosuppression. Clinical outcomes can then be compared across the subgroups, with association found for mortality after adjusting for known confounders in sepsis by different research teams [87] or worsening of a clinical score [90, 91].

Identified genes or other molecular features can be further investigated using established biological pathway datasets to understand their biological function. For example, recent work identified a poor outcome sepsis endotype, broadly replicated across different infectious disease contexts [86], that had features of maladaptive ‘emergency myelopoiesis’, with increased abundance of activated neutrophils, so-called myeloid-derived suppressor cells (MDSCs), haematopoietic stem and progenitor cells (HSPCs) and specific immature neutrophil populations. A patient’s membership in a subphenotype or endotype can be modelled as a continuous trait, rather than discrete categories [92] which increases the power to detect dynamic changes in immune status over the course of disease or in response to therapy. Conceptually, endotyping can also support the identification of existing or emerging animal model systems that best represent clinical CS subphenotypes to support preclinical testing [93–96].

The majority of such host response profiling has been performed in infectious diseases and sepsis where the relevance of peripheral blood is intuitive with a paucity of large-scale work performed using tissue samples of relevance to CS. The ShockOmics consortium contrasted leukocyte gene expression at three different time-points (days 1, 2 and 7) in 21 patients with septic shock and 11 patients with CS [97]. Patients were matched for demographics and illness severity and inclusion required survival at seven days which may have enriched for a cohort with a favourable trajectory. Overall gene expression features were found to be similar across the 2 shock sub-phenotypes. The major source of between-sample variation in this dataset corresponded to the time from disease onset to sampling, suggesting that patients with either septic shock or CS follow broadly similar host response trajectories from the first to the seventh day of intensive care unit admission.

As the most prevalent aetiology of CS, study of the ‘host response’ to AMI may provide insights into the pathobiology and heterogeneity of patients who progress towards CS. Hence, study of leukocyte gene expression in over 100 patients with AMI identified 2 sub-phenotypes which coded for proteins related to platelet function. Patients with sub-phenotype 2 exhibited higher CRP on admission than those with sub-phenotype 1. Gene set enrichment analysis of 139 patients in eleven datasets of PBMCs from AMI patients highlighted extensive changes characterised by pro-inflammatory activation and enhanced leukocyte-platelet interactions with one-third of patients classified into a hyper-inflammatory group [98]. Stratification by consensus clustering suggested AMI patients differ in the severity of inflammatory activation; however, there were no data to associate this with progression to or severity of CS. Speculatively, these data suggest that differential pathway activation may contribute to different CS sub-phenotypes. The Prospective Observational Study Investigating Genomic Determinants of Outcome From Cardiogenic Shock (GOlDilOCS, ClinicalTrials.gov Identifier: NCT05728359) and VANQUISH Shock [99] will analyse these associations at a gene expression and proteome level in prospective study of 300 and 600 CS patients, respectively.

Whilst conceptually appealing, the approach of whole blood sampling may not, however, be a relevant proxy measure for host response patterns in remote organs or cells [100]. In the context of CS, targeted collection of coronary endothelial or even myocardial/endocardial samples may be a useful addition to the more accessible peripheral blood samples which remain the mainstay of studies of the host response. Because these tissue samples are difficult to acquire, emerging modalities which provide information on damage occurring in remote organs are likely to be of interest.

One example of this is cell-free DNA methylation sequencing that is a developing technique which can provide information on damage occurring in remote tissues [101]. This extracellular DNA is released by dying cells and then passes into the peripheral blood compartment, where it circulates with a half-life of 1–2 h. DNA methylation patterns are tissue-specific, in most instances due to conserved epigenetic enhancer usage across cell types. Sequencing and deconvolution of the circulating cell-free DNA provides an indirect index of the degree of cell death occurring in organs which are difficult to sample. This approach may complement the use of existing clinical tests for organ-specific damage in CS such as cardiomyocyte troponin and hepatocellular aminotransferases. It also offers an opportunity to investigate the inconsistently observed association between increased levels of circulating cell-free DNA and adverse outcomes in critical illness [102–104], which could be related to differences in the tissue-of-origin of the circulating DNA. Proof-of-concept studies have demonstrated cardiomyocyte-specific release in heart failure [105] and after MI [106]. Investigation of the dynamic release of cfDNA specifically derived from the vascular endothelial cells (VECs) of different organs demonstrated that organ-specific VEC cfDNA can be detected in plasma during clinical illness—for example, lung-derived VEC dfDNA during sepsis, exacerbations of chronic respiratory disease and after cardiac catheterisation [107].

Cell-free DNA can be readily isolated from frozen plasma samples and can be amplified inexpensively for a small number of cell-type defining methylation sites or sequenced *in toto* to assess for a wide range of cell-type-specific patterns. As this method matures, it is conceivable that this may become a useful tool for dynamic assessment of organ function, for example before and after administration of a drug or initiation of MCS support in CS. This could be of particular interest in CS, where understanding the effect of tissue hypoperfusion on specific organs is likely to be a useful tool for patient phenotyping.

### Parallels with other critical illness syndromes: exotyping

Given the absence of therapies that improve outcome in CS and the observation that restoration of the low cardiac output that is the *conditio sine qua non* for CS has not improved clinical outcomes [108, 109], it serves to reason that the current concepts of CS may not adequately capture the complexity of what is essentially a critical illness syndrome.

Critical illness states such as sepsis and CS have historically been defined by the primary organ dysfunction combined with constellations of non-specific clinical, biochemical and physiological abnormalities. It is apparent that many of these abnormalities are shared across critical illness syndromes regardless of the initial insult [10, 22]. Whilst there is clearly heterogeneity both within critical illness syndromes and between them, this apparent homogeneity raises the prospect that despite disparate insults, similar underlying biological mechanisms or molecular signatures exist across critical illness syndromes as drivers of a common physiological derangement. This is the concept of exotypes, defined as endotypes conserved across different syndromes and sub-phenotypes [26].

Hence, the genomic response to trauma, severe burns and endotoxin exposure differed largely only in the duration rather than the response itself [110, 111]. The observed transcriptional up-regulation of inflammatory mediators mirrors that in ARDS and pancreatitis [111]. Comprehensive, longitudinal immune profiling in patients with infectious (sepsis) and sterile (traumatic injury and post-surgery) injury demonstrated common signatures of pro-/anti-inflammatory, innate and adaptive immune responses [62]. Whilst coronary ischaemia–reperfusion will be exclusive to AMI-CS, the pathobiological drivers of the systemic endothelial dysfunction and organ dysfunction observed in sepsis may be contributory in CS. For example, perturbations of the angiopoietin-2 -Tie 2 axis, a regulator of capillary permeability, have been observed in both sepsis [112, 113] and AMI-CS [114] with elevations of angiopoietin-2 associated with both poor outcome and coronary reperfusion success. It is therefore conceivable that other mechanistic drivers of sepsis, namely immune activation/dysfunction, mitochondrial dysfunction, complement activation, renin–angiotensin–aldosterone system activation and microcirculatory dysfunction [115–117], represent sub-phenotypes of AMI-CS. The identification and validation of biomarkers of AMI-CS sub-phenotypes, such as bio-adrenomedullin for severe or refractory vasoplegia [118], raises the enticing prospect of future targeted therapies across a range of shock states including AMI-CS. Future multi-omic preclinical and clinical study should be undertaken to identify conserved molecular responses across sub-phenotypes of differing shock states, specifically those patients with refractory shock who appear to be at highest risk of death (Graphical Abstract).

## Future directions

### Precision medicine in cardiogenic shock

**Fig. 2:**
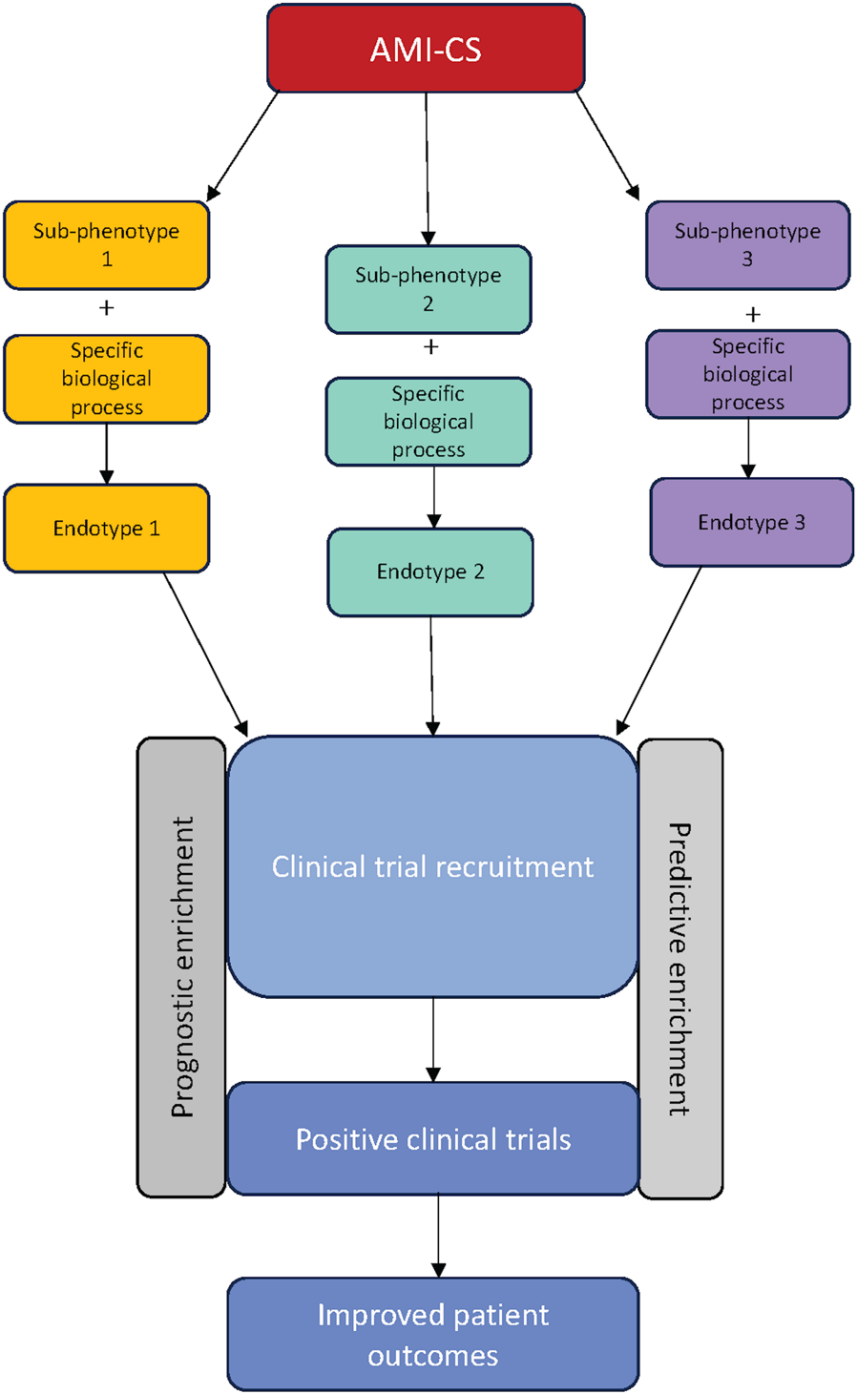
Endotype enrichment of future clinical trials in AMI-CS. AMI-CS = Acute myocardial infarction cardiogenic shock. Future research into the host response to cardiogenic shock should aim to identify endotypes which can then be used to enrich clinical trials to increase the likelihood of positive trial results

Secondary analyses from prior clinical trials in CS have failed to identify subgroups with clear differential treatment effects. This may reflect limitations in conventional one-at-a-time subgroup analysis which may be mitigated by machine learning methods such as causal forests or risk-based modelling, as recently demonstrated [119–121]. This may also reflect the choice to partition based on clinical groups as opposed to either molecular sub-phenotypes or endotypes described herein. Whilst advances continue to be made in clinical phenotyping of CS, greater precision, specifically linking clinical traits with pathobiology, is required to identify populations who will predictably respond to existing or emerging therapies. The identification of CS endotypes and treatable traits [122] offers the potential to enrich future clinical trial design and interventions through a more personalised or precision medicine approach (Fig. 2).

An endotype or treatable trait could be incorporated into a clinical trial as either a stratifying variable at recruitment, or in the case of dynamic and especially quantitative endotype systems, as a response measure. The first scenario is oftentimes divided into ‘prognostic’ and ‘predictive’ enrichment [123]. In prognostic enrichment, a set of predictors thought to have prognostic value, generally for mortality, are used as a screening mechanism at trial recruitment. Limiting recruitment to the cohort at highest risk of adverse outcome leverages the assumption that individuals with the most extreme risk may experience differential treatment effects. This assumption should be subject to some scrutiny given how frequently the opposite interpretation, that a trial has returned negative results because its population is too unwell to have the possibility to benefit (e.g. later-stage, unmodifiable risk), is also offered in the literature [124]. Risk-based heterogeneity of treatment response may be nonlinear, with greatest benefit in those at sufficient risk of the outcome to benefit, but not so sick as to be past the point where treatment is beneficial. Nonetheless, recent work in sepsis has demonstrated the potential for endotype-based stratification and quantitative scoring of patients with acute infection at point of care [92] by providing a framework that can be used with existing rapid turnaround methods (real-time quantitative reverse transcription PCR, qRT-PCR), as well as full-transcriptome technologies. This opens up the potential for future bedside, prognostic enrolment into clinical trials and even personalized therapeutic decision-making.

An alternative endotype-based strategy is so-called predictive enrichment, where a measurable trait, thought to have a biological relationship with experimental treatment, is used to select patients with a higher expected likelihood of benefit. Endotype stratification has been used in post hoc analyses of sepsis clinical trials. Whilst the VANISH randomised controlled trial [125] showed no mortality effect associated with corticosteroid treatment in sepsis, an interaction was found between sepsis endotype at baseline and mortality, with patients assigned to the lower-risk ‘immunocompetent’ endotype shown to have poorer survival [85]. Similar results were produced in a separate re-analysis using a separate endotype classification [126]. These associations are yet to be tested prospectively but suggest a route for developing endotype assignments as a stratifying factor for clinical trial design. Although the initial trial showed no mortality benefit with the use of polymyxin B haemofiltration versus sham [127], the post hoc division of patients by endotoxin activity showed differential effects on ventilatory-free days, mean arterial pressure (MAP) and mortality [128]. A large ongoing sepsis/ARDS trial is using 2 endotype classifications as pre-specified randomisation strata [129], will test these tools in the prospective setting.

The major challenge is finding an appropriate stratifying tool; to a large extent, traits which robustly predict treatment response are unknown before they are tested in trials. Post hoc stratification of trials with broad recruitment criteria can provide clues, but findings should be replicated in additional cohorts and ideally in preclinical model systems [130].

Caution should be exercised when estimating the likelihood that a null overall treatment response is masking unobserved heterogeneity of treatment effect across the trial population, and that high-dimensional assays can consequently be used to distinguish individual ‘responders’ from ‘non-responders’. Most applications in critical care allow only a single measure of treatment response for each patient [131]. When observing statistical heterogeneity in a response variable after a single episode of treatment, this may potentially be explained by statistical noise rather than a true biological difference and can easily be compounded by arbitrary dichotomization of a continuous variable to distinguish ‘responders’ from ‘non-responders’ [132]. The often-made statement that null overall treatment responses in critical illness therapeutic trials could reflect unmeasured heterogeneity in either the clinical phenotype or in the treatment response should be tested, not simply assumed to be true [133]. Emerging applications of adaptive clinical trial designs are being deployed in acute and critical illnesses that may be more amenable than conventional designs to the prospective identification of heterogeneity of treatment effect [134].

## Conclusion

New therapies and new approaches to clinical trial design are an urgent and unmet need if we are to improve the current lethality of CS. AMI-CS is increasingly recognised as encompassing features of systemic inflammation in addition to the systemic hypoperfusion due to pump failure. The holy grail of CS management is a granular understanding of disease heterogeneity as it relates to specific disease mechanisms and physiologic responses that would afford the opportunity to identify bespoke treatments with predictable responses that improve patient outcomes. Using the more nuanced approaches outlined herein may offer insights into the underlying molecular mechanisms of AMI-CS, with potential parallels to other critical illness syndromes and allow a transition from the current risk-based approach towards a mechanistic approach that embraces the heterogeneity within the CS population.

## Abbreviations

ACS: Acute coronary syndrome
AMI: Acute myocardial infarction
ARDS: Acute respiratory distress syndrome
CICU: Cardiac critical care unit
CO: Cardiac output
CS: Cardiogenic shock
cfDNA: Cell-free DNA
cDPP3: Circulating dipeptidyl peptidase
CH: Clonal haematopoiesis
CRP: C-Reactive protein
DNA: Deoxyribonucleic acid
DPP3: Dipeptidyl peptidase 3
ECLS: Extra-corporeal life support
ECMO: Extra-corporeal membrane oxygenation
G-CSF: Granulocyte-colony-stimulating factor
GDF-15: Growth differentiation factor-15
HSC: Haematopoietic stem cells
HSPCs: Haematopoietic stem and progenitor cells
HF: Heart failure
HTE: Heterogeneity of treatment effect
IFN-g: Interferon gamma
ICU: Intensive care unit
IL-1: Interleukin-1
IL-1b: Interleukin-1 beta
IL-1Ra: Interleukin-1 receptor antagonist
IL-5: Interleukin-5
IL-6: Interleukin-6
IL-7: Interleukin-7
IL-8: Interleukin-8
IL-10: Interleukin-10
MIP-1b: Macrophage inflammatory protein-1 beta
MCS: Mechanical circulatory support
MCP-1: Monocyte chemoattractant protein-1
MCP-1b: Monocyte chemoattractant protein-1 beta
MOF: Multi-organ failure
MDSCs: Myeloid-derived suppressor cells
MI: Myocardial infarction
NLR: Neutrophil-to-leucocyte ratio
NSTEMI: Non-ST elevation myocardial infarction
NT-pro-BNP: N-terminal pro-brain natriuretic peptide
PTX 3: Pentraxin 3
PCI: Percutaneous coronary intervention
PBMCs: Peripheral blood mononuclear cells
Prdx-1: Peroxiredoxin-1
PLR: Platelet-to-lymphocyte ratio
PCAS: Post-cardiac arrest syndrome
PCT: Procalcitonin
RANTES: Regulated on activation, normal T cell expressed and secreted
RNA: Ribonucleic acid
SelP: Selenoprotein P
SS: Septic shock
SCAI: Society for Cardiovascular Angiography and Interventions
STEMI: ST-elevation myocardial infarction
TET2: Tet methylcytosine dioxygenase 2
TNF-a: Tumour necrosis factor alpha
US: United States
VECs: Vascular endothelial cells
WBCs: White blood cells
